# Supplementary material for: Microtubule damage shapes the acetylation gradient
Source: Nat Commun. 2024 Mar 6;15:2029. doi: 10.1038/s41467-024-46379-5 (PMC10918088; doi:10.1038/s41467-024-46379-5)
Supplement: Supplementary file 1 — Supplementary Information [file 41467_2024_46379_MOESM1_ESM.pdf]

SUPPLEMENTARY INFORMATION

SUPPLEMENTARY FIGURES

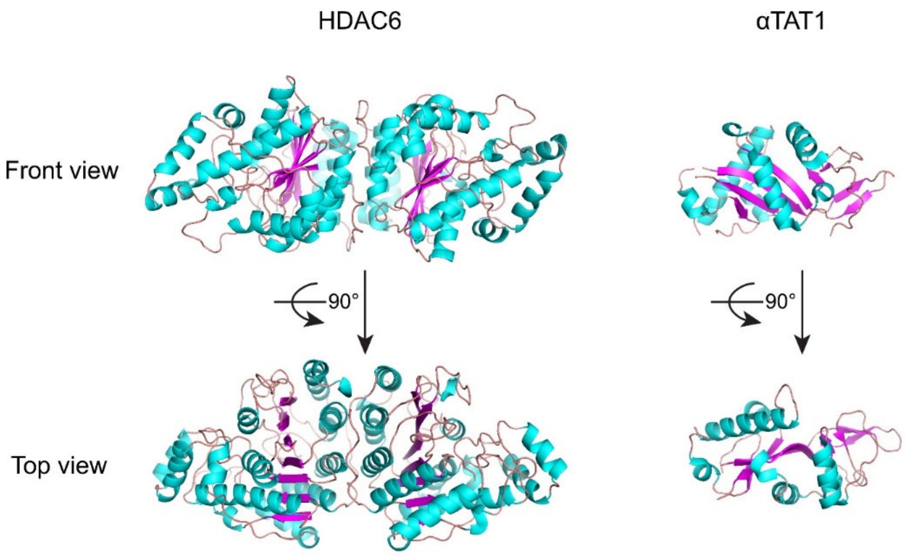

Supplementary Fig. 1. Representation of the structured domains of HDAC6 and αTAT1. Front and top view of the AlphaFold prediction of the structured HDAC6 catalytic domain (AF-Q9UBN7) [https://alphafold.ebi.ac.uk/entry/Q9UBN7] (left). Front and top view of the αTAT1 structured region (4GS4) [https://doi.org/10.2210/pdb4GS4/pdb] (right). α-helices are colored in blue and β-strands in pink. Note that both proteins have predicted unstructured domains which are not represented here. Structures were adapted using Pymol.

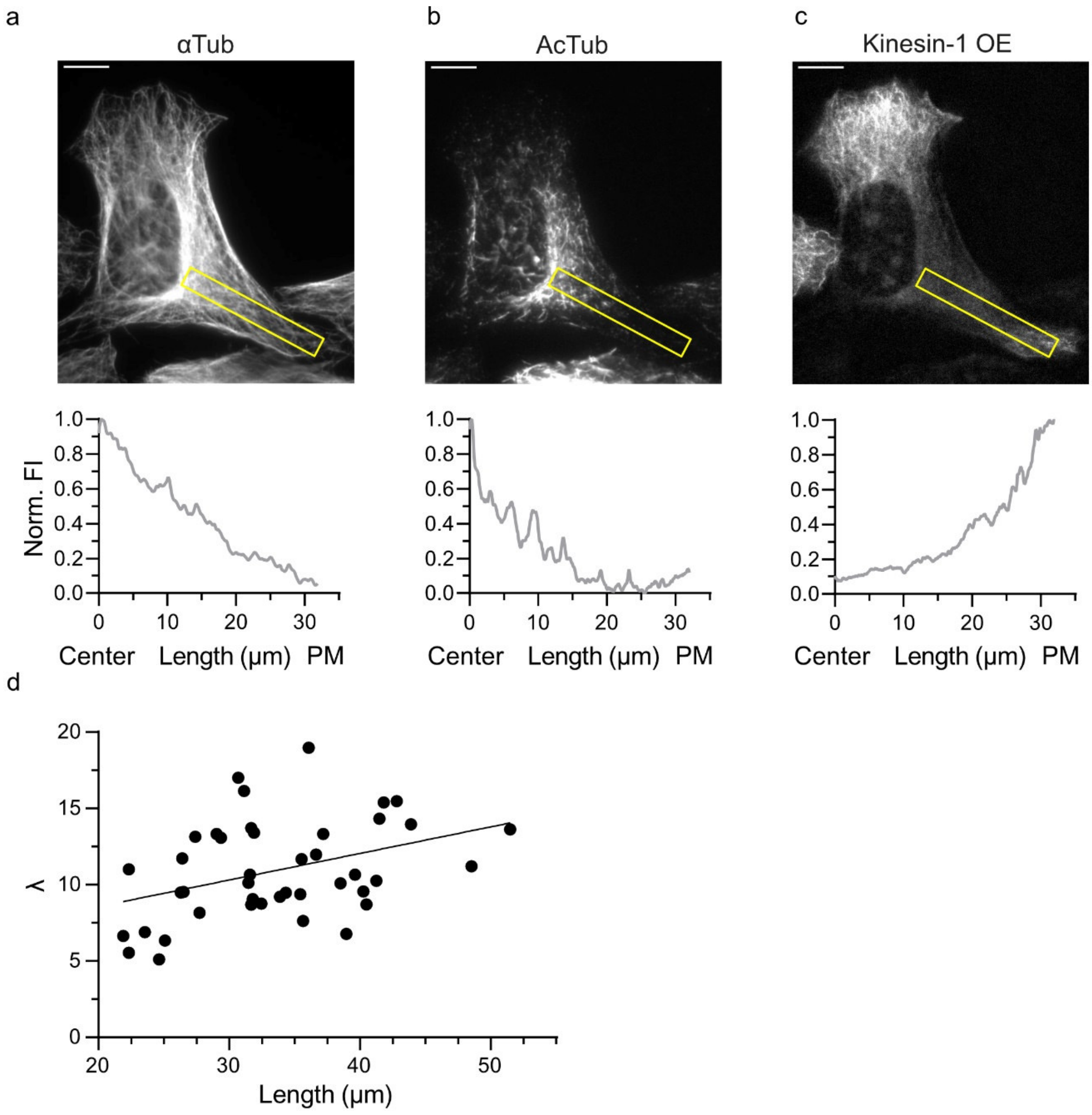

Supplementary Fig. 2. Characteristics of the microtubule acetylation array in HeLa cells. **a**, **b** and **c**, Representative cell with selected 45-pixel line for analysis of the fluorescence intensity profile of the microtubule network with corresponding intensity profile normalized to the maximum intensity value (**a**), acetylated tubulin array (**b**) and kinesin-1 overexpression (**c**). The minimum value of acetylated tubulin was set to 0. Scale bars: 10 µm. **d**, Correlation of the characteristic length λ of the single exponential fits and the cell length from the nucleus to the plasma membrane in HeLa WT cells (n= 42 cells) from 3 independent experiments from Fig. 2c. Data were fitted using a simple linear regression (R^2 = 0.15) using GraphPad Prism software v.9. Source data are provided as a Source Data file.

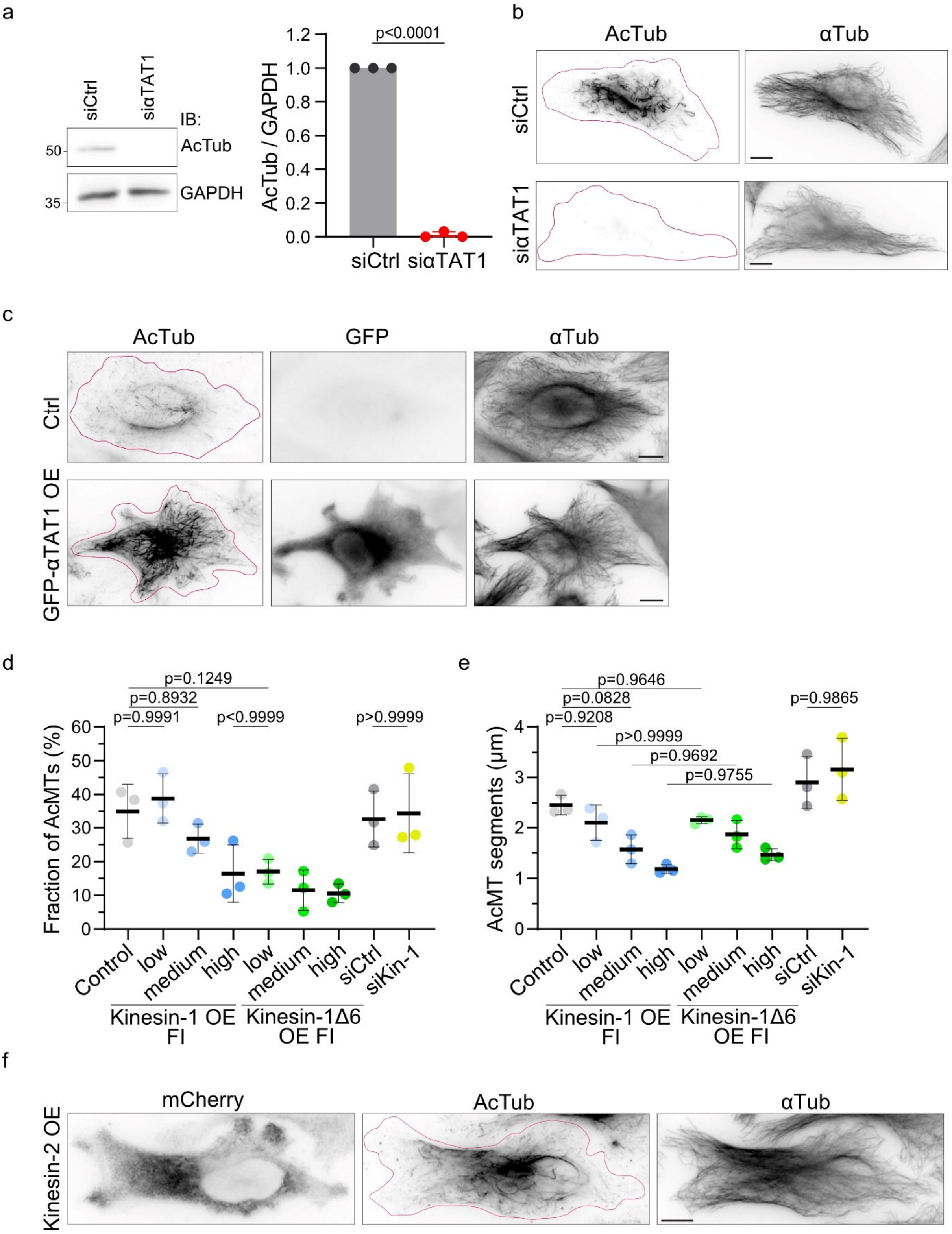

Supplementary Fig. 3. Acetylation levels depend on the activity of αTAT1 and kinesin-1 variants but not kinesin-2. a, Representative western blot (left) with quantification of AcTub levels relative to GAPDH (right) in HeLa control cells (siCtrl) and αTAT1 knock-down (siαTAT1) cells from 3 independent experiments. Statistics: two tailed t test. Mean with SD. **b**, Representative immunofluorescence images of HeLa siCtrl and siαTAT1 cells. **c**, Representative immunofluorescence images of HeLa cells overexpressing GFP or GFP-αTAT1 and stained for AcTub and αTub. The magenta outline defines the edges of the cells. **d** and **e**, Means with SD for the 3 independent experiments of Fig. 2e (**d**) and Fig. 2f (**e**). Statistics: one-way ANOVA. **f**, Representative

immunofluorescence images of HeLa cells overexpressing KIF17-mCherrry (Kinesin-2 OE) and stained for AcTub and αTub. The magenta outline defines the edges of the cells. Scale bars: 10 µm. Source data are provided as a Source Data file.

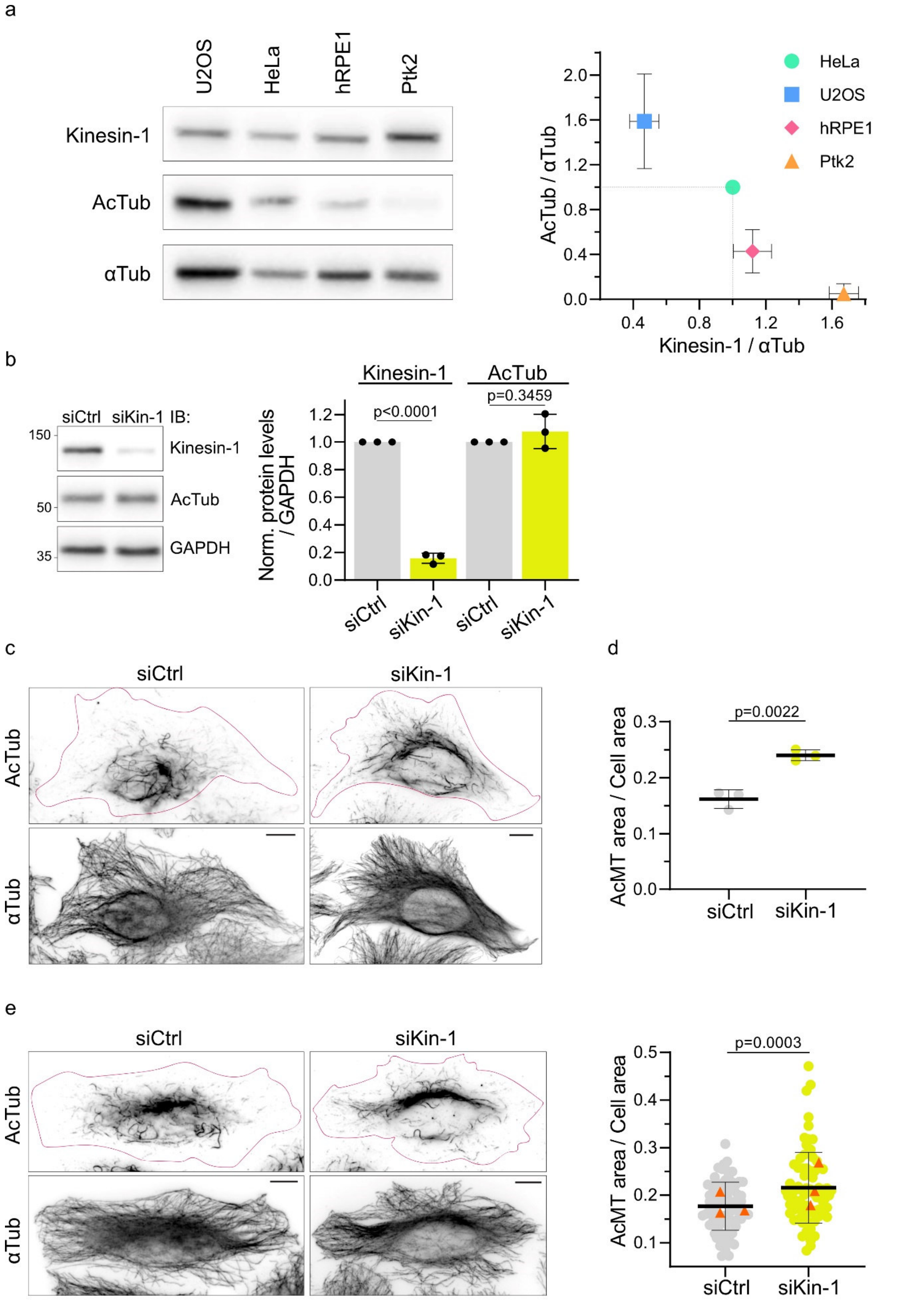

Supplementary Fig. 4. Impact of kinesin-1 on microtubule acetylation. a, Representative western blot (left) with quantification of the levels of kinesin-1 and AcTub relative to αTub (right) in different cell lines from 3 to 5 independent experiments. Relative kinesin-1 and AcTub levels were normalized to the HeLa cell values. Mean

with SD. **b**, Representative western blot analysis with quantification of kinesin-1 and AcTub levels relative to GAPDH from 3 independent experiments. Statistics: two tailed t test. Mean with SD. **c**, Representative immunofluorescence images of HeLa siCtrl (left) and siKin-1 (right) stained for AcTub and αTub. **d**, Means with SD for the 3 independent experiments from Fig. 2g. Statistics: two tailed t test. **e**, Representative immunofluorescence images of HeLa kinesin-1 knock-down and control cells with the quantification of the area of acetylated microtubule array / total cell area in siKin-1 (n = 76 cells) compared to control cells (n = 76 cells) from 3 independent experiments. Statistics: two tailed t test. Mean with SD. Means for each independent experiment are shown as orange triangles (p=0.2603). Statistics: two tailed t test. Cells were stained for AcTub and αTub. The magenta outline defines the edges of the cells. Scale bars: 10 µm. Source data are provided as a Source Data file.

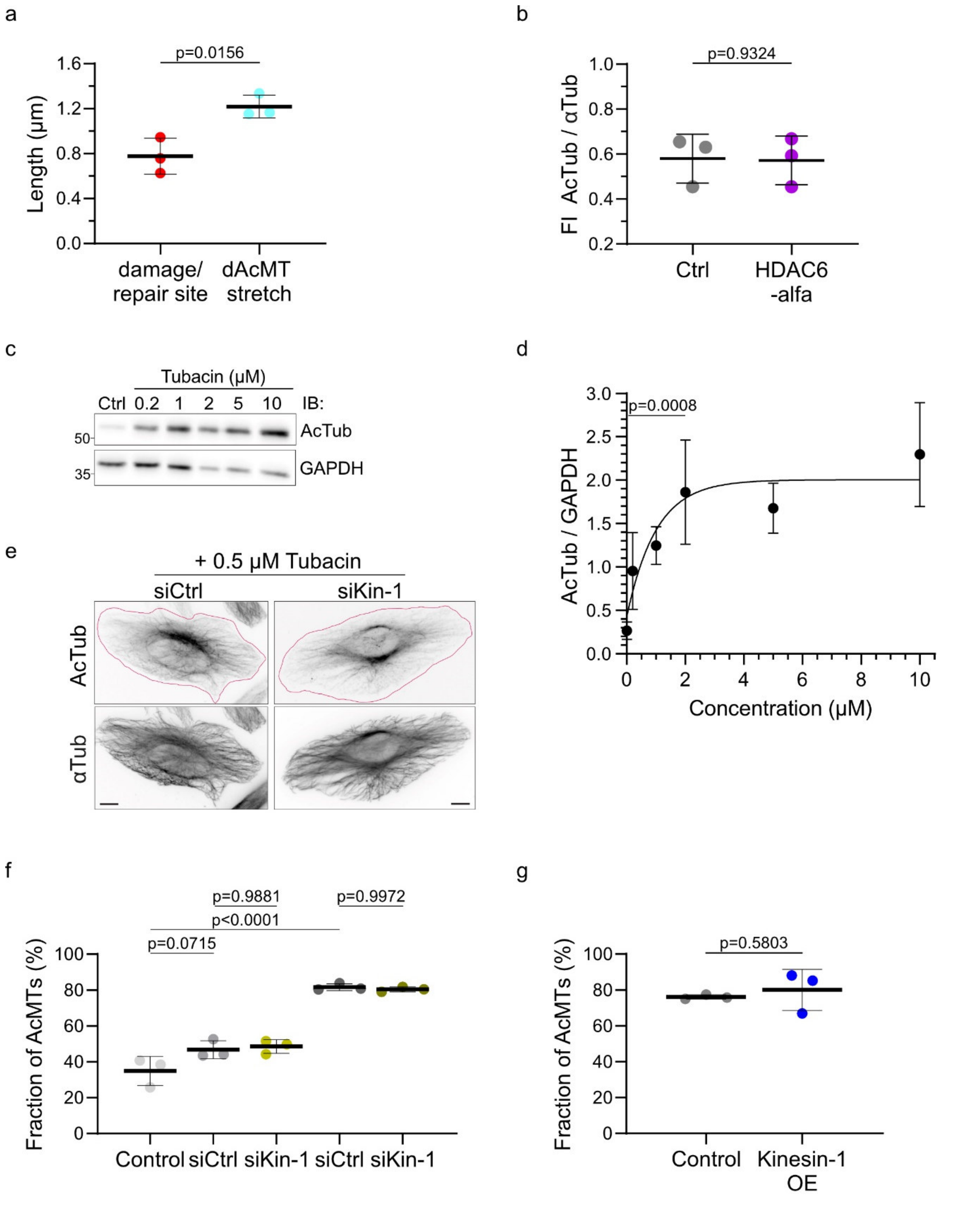

Supplementary Fig. 5. Tubacin increases tubulin acetylation in a dose-dependent manner. Changes in acetylation levels after HDAC6 and kinesin-1 overexpression, titration of Tubacin and kinesin-1 knockdown. a and **b,** Means with SD for 3 independent experiments quantified in Fig. 3g (**a**) and Fig. 4a (**b**). Statistics: two tailed t test. **c** and **d,** Representative western blot analysis (**c**) with quantification (**d**) of AcTub levels relative to GAPDH in HeLa WT cells after 60 min in presence of 0.2, 1, 2, 5 and 10 µM Tubacin, from 3 to 4 independent experiments. Statistics: two-way ANOVA. Mean with SD. **e,** Representative immunofluorescence images of HeLa cells, control cells (siCtrl) and kinesin-1 knock-down (siKin-1), both siRNA conditions were treated with 0.5 µM Tubacin for 1h at 37°C before fixation. Cells were stained for AcTub and αTub. The magenta outline defines the edges of the

cells. Scale bars: 10 µm. **f** and **g,** Means with SD for 3 independent experiments quantified in Fig. 5c (**f**) and Fig. 5d (**g**). Statistics: one-way ANOVA (**f**), two tailed t test (**g**). Source data are provided as a Source Data file.

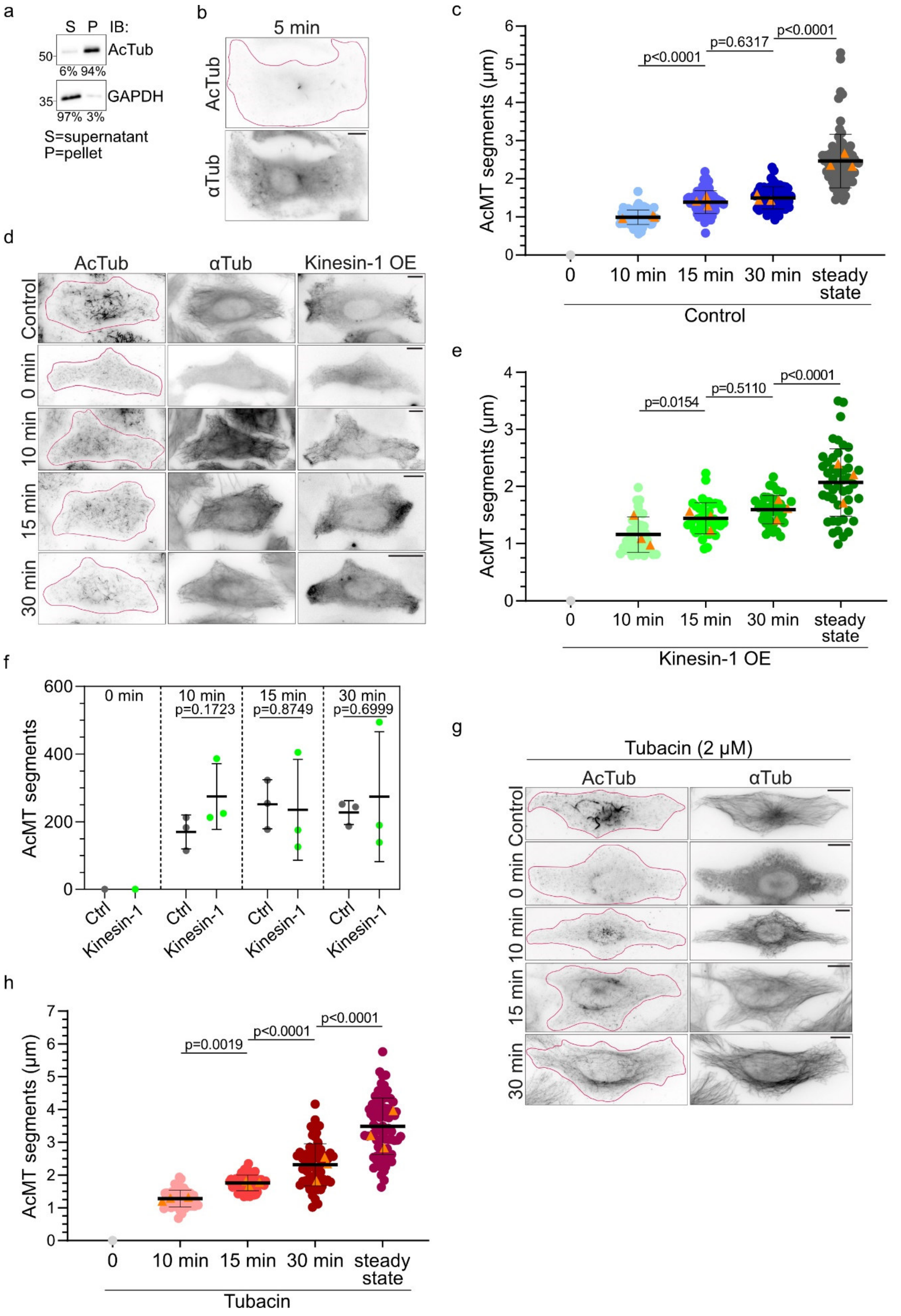

Supplementary Fig. 6. Establishment of microtubule acetylation during network regrowth. a, Representative western blot of the fraction of AcTub present in the cytosol (S) and polymerized within the microtubule network (P) in HeLa cells. The cytosolic protein GAPDH was used to validate the purity of the pelleting assay. **b**,

Representative immunofluorescence images of microtubule regrowth (5 min) after cold treatment and stained for AcTub and αTub. **c**, Quantification of the average AcTub stretch size in HeLa WT cells after 0, 10 (n = 63 cells), 15 (n = 66 cells) and 30 min (n = 61 cells) of microtubule regrowth or steady state conditions (n = 80 cells). Means for each independent experiment are shown as orange triangles (p=0.0197 (10-15), p=0.9431 (15-30), p<0.0001 (30-steady)). Statistics: one-way ANOVA. Mean with SD. **d,** Representative immunofluorescence images of a control cell and microtubule regrowth (0, 10, 15 and 30 min) after cold treatment in cells overexpressing K560 and stained for AcTub and αTub. **e**, Quantification of the average AcTub stretch size in HeLa WT cells overexpressing K560-GFP after 0, 10 (n = 38 cells), 15 (n = 40 cells) and 30 min (n = 36 cells) of microtubule regrowth or steady state conditions (n = 45 cells). Means for each independent experiment are shown as orange triangles (p=0.7802 (10-15), p=0.9123 (15-30), p=0.2086 (30-steady)). Statistics: one-way ANOVA. Mean with SD. **f**, Means with SD for 3 independent experiments from Fig. 6e. Statistics: two tailed t test. **g,** Representative immunofluorescence images of a control cell and microtubule regrowth (0, 10, 15 and 30 min) after cold treatment in the presence of 2 µM Tubacin and stained for AcTub and αTub. **h**, Quantification of the average AcTub stretch size in HeLa WT cells in the presence of 2 µM Tubacin after 0, 10 (n = 48 cells), 15 (n = 40 cells) and 30 min (n = 62 cells) of microtubule regrowth or steady state conditions (n = 76 cells). Means for each independent experiment are shown as orange triangles (p=0.4779 (10-15), p=4923 (15-30), p=0.0281 (30-steady)). Statistics: one-way ANOVA. Mean with SD. Scale bars: 10 µm. The magenta outline defines the edges of the cell. Source data are provided as a Source Data file.

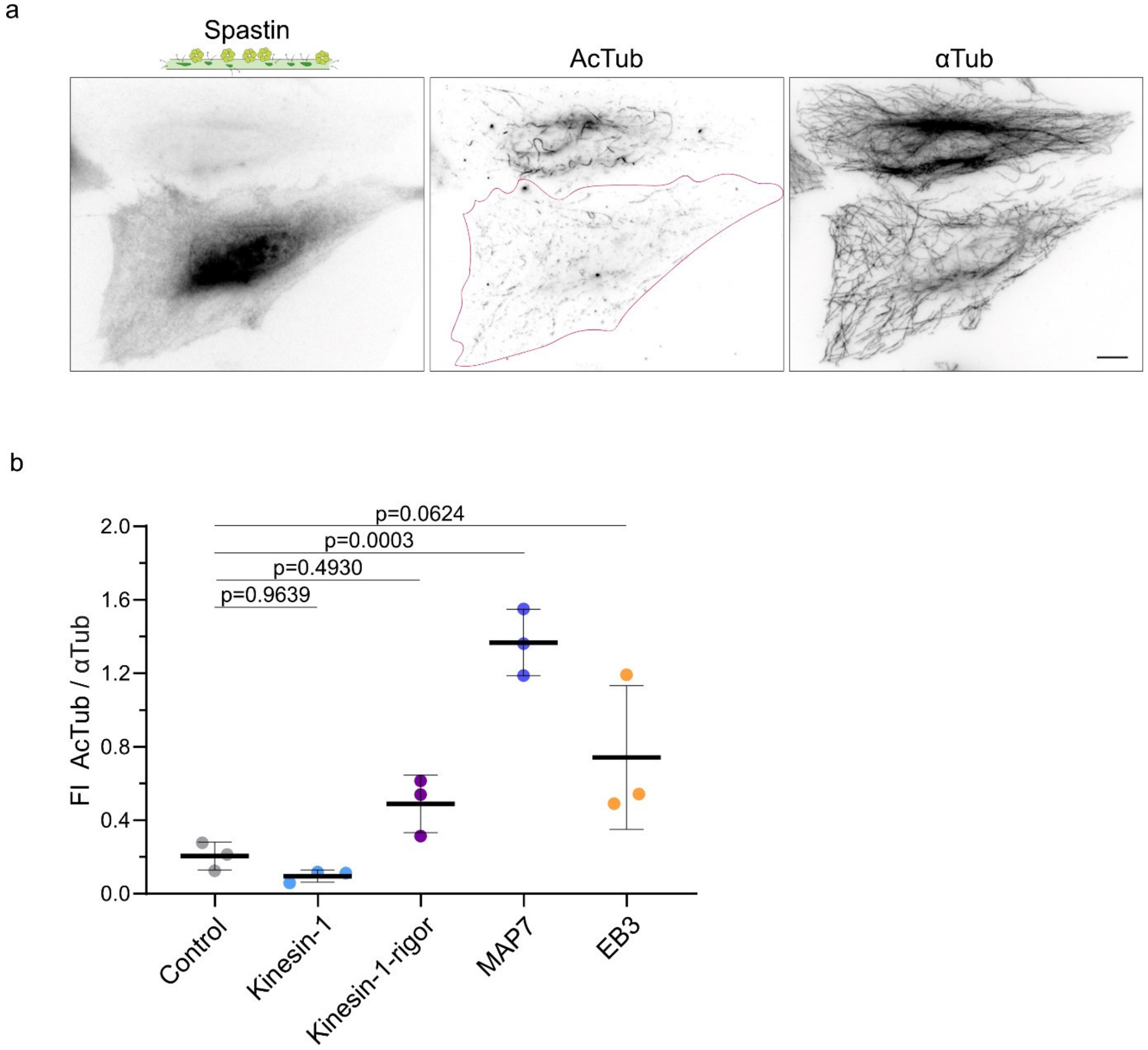

Supplementary Fig. 7. High levels of Spastin overexpression result in a fragmented microtubule network. a, Representative immunofluorescence of HeLa cells transfected with mCherry-Spastin. Cells were stained for AcTub and αTub. The magenta outline defines the edges of the cell overexpressing Spastin. Scale bar: 10 µm. **b**, Means with SD for 3 independent experiments from Fig. 7b. Statistics: one-way ANOVA. Source data are provided as a Source Data file.
